# First Report on Ovine Paratuberculosis in the Sudan: Diagnosis Using Different Techniques

**DOI:** 10.3390/ani12233312

**Published:** 2022-11-27

**Authors:** Sanaa M. Idris, Eva A. Ali, Wisal A. Elmagzoub, Julius B. Okuni, Mohamed E. Mukhtar, Lonzy Ojok, ElSagad Eltayeb, Ahmed Abd El Wahed, Kamal H. Eltom, Ahmed A. Gameel

**Affiliations:** 1 Department of Animal Health and Safety of Animal Products, Institute for Studies and Promotion of Animal Exports, University of Khartoum, Shambat 13314, Khartoum North, Sudan; 2 Department of Pathology, Faculty of Veterinary Medicine, University of Khartoum, Shambat 13314, Khartoum North, Sudan; 3 Department of Biology and Biotechnology, College of Applied and Industrial Sciences, University of Bahri, Alkadaro 13311, Khartoum North, Sudan; 4 College of Veterinary Medicine, Animal Resources and Biosecurity (COVAB), Makerere University, Kampala P.O. Box 7062, Uganda; 5 Department of Agricultural Extension and Rural Development, Faculty of Agriculture, University of Khartoum, Shambat 13314, Khartoum North, Sudan; 6 Department of Pathology, Faculty of Medicine, Gulu University, Gulu P.O. Box 166, Uganda; 7 Department of Surgery, Faculty of Medicine, Al Neelain University, Almogran 11111, Khartoum, Sudan; 8 Institute of Animal Hygiene and Veterinary Public Health, Faculty of Veterinary Medicine, University of Leipzig, An den Tierkliniken 43, D-04103 Leipzig, Germany

**Keywords:** *Mycobacterium avium paratuberculosis*, sheep, recombinase polymerase amplification

## Abstract

**Simple Summary:**

Paratuberculosis (PTB) is a neglected disease in Africa, and it is a hidden killer disease of large and small ruminants with utmost socio-economic importance, especially for rural communities. The disease has been reported in the Sudan in both goats and cattle, but not in sheep. In this study we report the disease for the first time in sheep in the country. We suspected this disease in a breeding flock of sheep because of a history of unknown wasting condition. In this context, we used different diagnostic techniques to detect the bacteria causing the disease and to confirm the disease presence. As sheep in the Sudan are the most important export animal, it is worthy to conduct a wide investigation to gain insight into the disease prevalence in sheep to set up preventive measures and implement control programmes, as well as raising the awareness of animal owners in the country.

**Abstract:**

Paratuberculosis (PTB) has been reported in the Sudan in cattle and goats for more than 50 years but has never been reported in sheep. However, suspicion of the disease in a breeding flock of sheep in Khartoum North locality was made due to a history of unknown cause of loss of weight. Blood and faecal samples were collected from all animals (N = 59): harvested sera were tested for anti-*Mycobacterium avium* subsp. *paratuberculosis* (MAP) antibodies by Enzyme Linked Immunosorbent Assay (ELISA); faeces were screened for acid-fast bacilli by Ziehl–Neelsen staining, tested for MAP DNA by recombinase polymerase amplification (RPA) and some faecal samples were cultured for MAP isolation. Typical MAP acid-fast bacilli were seen in 10.2% (6/59) of the faecal smears, 37.5% of the tested faecal samples (12/32) were positive for MAP DNA and only 3 (5.1%) animals were seropositive for MAP. MAP positive cultures were obtained from 2 out the 6 samples showing typical MAP acid-fast bacilli; the isolates were confirmed by real-time PCR and sequencing. As sheep are animals of utmost economic importance as the main export animals for the country, this first report of ovine PTB warrants special considerations and more investigations for planning control programmes of the disease.

## 1. Introduction

Paratuberculosis (PTB) or Johne’s disease is a chronic infectious disease of ruminants and many non-ruminant animals of worldwide distribution [1,2,3]. It is caused by *Mycobacterium avium* subsp. *paratuberculosis* (MAP), which is able to survive in the environment and in the host tissue by virtue of its unique cell wall structure [4]. The disease causes serious economic losses related to mortality, early culling, reduction in productivity, costs of diagnosis, treatment, and control [5,6]. MAP is also of public health concern, as it has been suspected to cause Crohn’s disease (an idiopathic chronic inflammatory bowel disease) in humans, but this remains to be confirmed [7,8]. The primary mode of MAP transmission between animals is by ingestion of feed or water contaminated with faeces of infected animals. MAP can also pass from an infected dam to its foetus or after delivery by colostrum and milk [9,10].

Despite having been diagnosed in the Sudan in cattle and goats earlier [11,12,13], and MAP was isolated for the first time from goats in the country [14], reports on PTB in the Sudan are scarce, especially in small ruminants [15]. However, two recent seroprevalence studies on bovine PTB [16,17] suggested that the disease is widely spread among dairy cattle herds but has never been reported in sheep.

With an estimated population of 50 million heads [18], sheep in the Sudan are of the utmost economic importance, being the main export animal, and its meat is the most preferred by people in the country. Moreover, sheep play important socio-economic roles in rural communities, in addition to providing milk and meat for domestic consumption [19]. This role would be greatly reduced by diseases of chronic courses, such as PTB, which has been described as a hidden killer [15]. Here, we report on ovine PTB in the Sudan for the first time after suspicion of the disease in a breeding flock with a history of an unknown wasting condition. In this respect, we compared between microscopy, serology, culture, and molecular biology techniques for diagnosing the disease in the flock.

## 2. Materials and Methods

### 2.1. Animals

A flock of indigenous desert sheep composed of 59 heads (58 ewes and one ram) had been kept on a farm in Khartoum North locality for more than 10 years. The age of the animals ranged from 1 to 10 years and the body weight between 30 and 45 kg. Some ewes showed weight loss over time, according to the animal herder, with softening of the faeces. With the owner’s permission, from each animal, 4 mL blood samples were taken by jugular venipuncture in plain tubes, and 2–4 balls of faeces were collected into plastic containers. All samples were transported in ice boxes to the laboratory of the Institute for Studies and Promotion of Animal Exports, University of Khartoum. Serum was separated by centrifugation, and both sera and faecal samples were stored at −20 °C until needed.

### 2.2. Microscopic Examination

**Figure 1:**
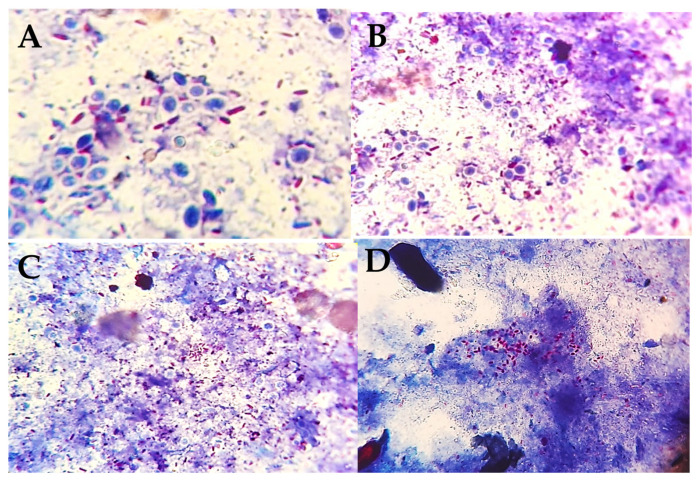
Arrangement of acid-fast bacilli in faecal smears (ZN ×1000). (**A**) Few scattered; (**B**) many scattered; (**C**) few clumps; (**D**) many clumps.

Smears made from a suspension of 1 piece of faeces in distilled water were stained by the Ziehl–Neelsen (ZN) method and examined microscopically for the presence of acid-fast bacilli. Smears showing short acid-fast bacilli scattered or grouped in clumps were considered positive. According to the distribution and density of the organism, 4 categories were recognized: few scattered, many scattered, few clumps, many clumps (Figure 1).

### 2.3. Enzyme-Linked Immunosorbent Assay (ELISA)

For the presence of anti-MAP-specific antibodies, the sera were investigated by an indirect ELISA using a commercial kit (*Mycobacterium paratuberculosis* Antibody Test Kit, IDEXX, Westbrook, ME, USA) for bovine and small ruminants according to the manufacturer’s instructions. The procedures included a pre-incubation step of the serum samples in a buffer containing *Mycobacterium phlei* to remove cross-reacting antibodies of other mycobacteria. Positive and negative results were determined according to the manufacturer’s instructions.

### 2.4. DNA Extraction

Extraction of MAP DNA from faecal samples as well as from cultures was done using a QIAamp DNA Blood Mini Kit (Qiagen, Hilden, Germany). Briefly, faecal suspension was prepared as mentioned above and centrifuged; the sediment was transferred to a Precellys tube (SK38, brown lid) to which 100 µL of PBS and 50 µL of 20% SDS were added and vigorously vortexed till homogenization was achieved. After incubation for 30 min at 37 °C, the lysis buffer was added with further incubation at 37 °C for 30 min. Then, 60 µL of proteinase K and 600 µL of lysis buffer were added with vortexing for 10 s. Incubation periods for 30 min and 15 min at 56 °C and 95 °C, respectively, were performed. The last step was followed by the addition of ethanol and then transfer of the sample to the spin column. Elution of the DNA from the column was achieved by 100 µL of the Buffer AE, incubated for 1 min at room temperature, and centrifuged at maximum for 5 min. The extracted DNA was stored at −20 °C until use in molecular assays.

### 2.5. Recombinase Polymerase Amplification (RPA)

A total of 32 faecal samples was selected for screening for MAP DNA using a real-time recombinase polymerase amplification (RPA) assay. The selection of the samples was as follows: all those showing many or few clumps of acid-fast bacilli (N = 6) and the rest from those showing scattered acid-fast bacilli by random selection. The assay used was as described previously [20] using a TwistAmp exo kit (TwistDX, Cambridge, UK) on the Mobile Suitcase Lab developed by Abd El Wahed et al. [21].

### 2.6. Real-Time Polymerase Chain Reaction (Real-Time PCR)

The real-time PCR assay described by Hansen et al. [20] was also used to detect MAP DNA in ZN positive cultures. The assay was performed in a total reaction volume of 20 μL containing Light Cycler 480 Probes Master Mix (Roche, Mannheim, Germany), molecular biology grade water, 10 μM of each primer and probe, and 5 μL DNA template. A modification of the annealing temperature to 55 °C was made.

### 2.7. Cultures of Faecal Samples

Cultures were made from faecal samples (N = 6) showing clumps of acid-fast bacilli in ZN-stained smears. The faecal samples were decontaminated using a sterile solution of 0.75% hexadecylpyridinium chloride (HPC) following standard decontamination protocols before being cultured in Middlebrook 7H11 agar (Merck, Darmstadt, Germany) slants supplemented with oleic acid–albumin–dextrose–catalase (OADC) and mycobactin J (2 mg/L, Allied Monitor, USA). The cultured slants were incubated at 37 °C for 4 weeks and then checked for growth every month. Visible growth was checked by ZN staining and confirmed by real-time PCR.

## 3. Results and Discussion

**Figure 2:**
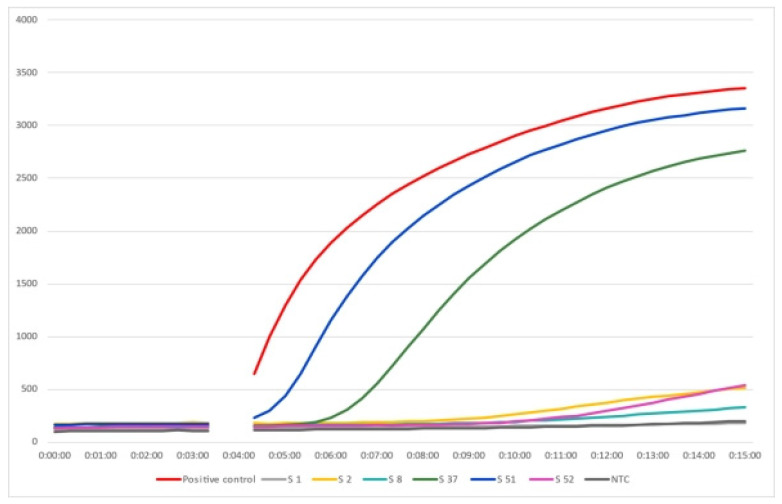
Amplification plot of the RPA assay for the detection of *Mycobacterium avium* subsp. *paratuberculosis* in sheep faecal samples.

Ovine paratuberculosis is a non-notifiable disease that has been reported in many countries [22] and is neglected in Africa and considered to be the same in other animals [23,24]. Owing to different pathological forms of the disease and the corresponding immune and cellular profiles [25], beside the compromised sensitivity and specificity of available diagnostics [26], various tests are required to diagnose the infection. In the current study, the disease was investigated using different tests in a small sheep breeding flock. PTB was suspected in this flock because of a history of loss of weight over time. As the flock was small, faeces of all animals were screened by Ziehl–Neelsen staining, and 98.3% (58/59) showed acid-fast bacilli, 10.2% (N = 6) of which showed arrangements of bacilli in clumps highly suggestive of MAP. Although this is a presumptive diagnosis depending on identifying the acid-fast bacilli arrangement (Figure 1), ZN staining has the advantage of being simple, rapid, and inexpensive [26]. Therefore, further investigations were conducted. The ELISA detected antibodies against MAP in only 5.1% (N = 3) animals. However, the sensitivity of ELISA is under question. Hope et al. [27] estimated the specificity and sensitivity of absorbed ELISA in diagnosing ovine PTB as 98.2 to 99.5% (CI) and 35 to 54% (CI), respectively. The low sensitivity of ELISA could also be attributed to weak and late antibody responses in sheep [28]. During the latent period of the disease, where MAP is not set free from macrophages to stimulate the immune response, antibody titres fluctuate as an animal progresses from the infected to the infectious to the diseased state [29,30]. Due to the variable immune and cellular responses, molecular assays were developed and found superior to immunodiagnostics. The sensitivity of molecular assays has improved over time; in one study, real-time PCR from faecal samples was proved to be more sensitive than culture (the gold standard) from intestinal tissues [25]. Therefore, more emphasis was given to the detection of MAP DNA, which was detected in 37.5% of the animals tested (12 out of 32). Selection for RPA was based on categorization of the arrangement of the acid-fast bacilli in the ZN- stained smears. All six samples that showed clumps of bacilli were positive for MAP DNA (Figure 2), confirming the presumptive diagnosis based on the ZN staining. However, 50% of the smears showing many scattered acid-fast bacilli were also positive for MAP DNA compared with 12.5% of those showing few scattered bacilli (Table 1). The RPA assay used here had 100% specificity and 89.5% sensitivity [20]. Thus, laboratory diagnosis or confirmation of ovine PTB would be more appropriate when detection of MAP DNA in the faeces is possible (Table 2). 

**Figure 3:**
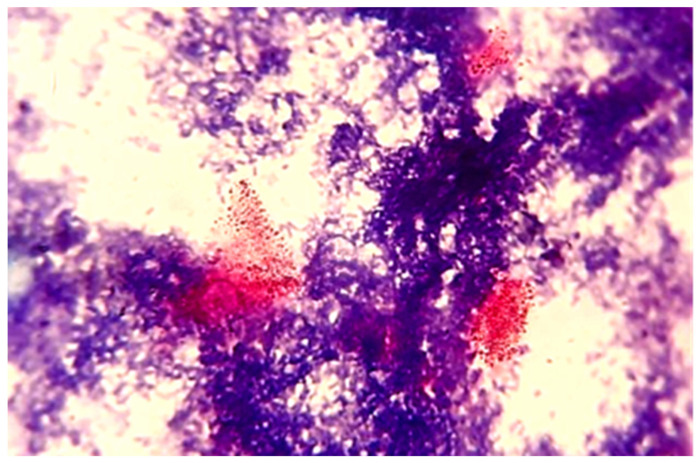
Acid-fast bacilli from *Mycobacterium avium* subsp. *paratuberculosis*-positive faecal culture (ZN ×1000).

Diagnosis of PTB by isolating MAP by culture is the “gold standard” [15]. However, Coelho et al. [31] stated that the sensitivity of MAP culture from faeces and tissue is less when compared with molecular methods and histopathology to confirm PTB in clinically diagnosed animals. He made this statement because he isolated MAP from 2 out of 30 (6.7%) sheep with PTB. The isolation of MAP by culture is difficult because of intermittent shedding of the bacteria and the low number of bacilli in faeces and tissues [32,33]. To overcome this difficulty in our investigation, we selected for culture the samples that showed clumps (many or few) of acid-fast bacilli (Table 1). Despite this, we obtained MAP-positive cultures from only two of these six (33.3%) cultured faecal samples (Figure 3). These isolates were confirmed by real-time PCR and partial sequencing of the IS 1113 (data not shown). However, our experience with human faecal cultures for MAP made at the same time is in total contrast to these findings. We found that culture is more sensitive than DNA detection by real-time PCR for MAP in human faecal or tissue samples [34]. 

The difference in sensitivity of cultures between human and sheep samples is probably due to difference between the strains; therefore, typing the human and sheep isolates that we have obtained is under investigation in a separate study.

Sheep is one of the major animal resources in the Sudan, which comprise about 20% of the country’s Gross Domestic Product (GDP). Most sheep breeding is practiced by small scale producers in rural areas, thus supporting the livelihood of these people. These producers bring their animals intended for sale to central rural markets on certain days of the week. In these markets, flocks from different areas come into contact, and risks of transmission of infectious diseases and parasites increase, which ultimately affects the economy of these producers as well as that of the country. Paratuberculosis is one of the most important ruminant diseases and has no treatment yet; it also causes great economic losses in terms of productivity and fertility/reproductivity of the animal. Once it is established, it is very difficult to get rid of. Most, if not all, bacterial diseases affecting small ruminant production in the Sudan have been largely controlled through proper diagnosis, treatment, and vaccinations. Unfortunately, PTB remains neglected despite very early reports about this disease [11,12,13,14]. This could be due to poor awareness about the disease, lack of guiding clinical signs (such as shooting profuse diarrhoea in bovines), the unavailability of improved/highly sensitive diagnostic assays [15,22,35], and the difficulty in recognizing latent and sub-clinical disease, especially in open pasture; however, the movement of animals in the range system with the possibility of passing large amount of faeces in the resting area at night before starting grazing the next morning seems to help in decreasing the chances of being infected. In addition, in lush pastures, sheep graze the top grass, lessening the possibility of contact with faeces. Moreover, the high ambient temperature and dryness in the open pasture may affect MAP survival. Therefore, it seems that the diagnosis of PTB in animals in enclosed areas or farms is more likely than in open pasture. This depends on clinically infected animals shedding the organism. Nevertheless, exported sheep are usually males of young age, i.e., they are not exposed to MAP long enough to show signs of clinical infection. In the case of a sheep-raising country such as the Sudan, negligence of ovine PTB and its prevalence would have irreversible future effects on the sheep population and economy in the country. Thus, assessment of the impact of the disease and control will be hampered by the lack of reliable and realistic epidemiological data and the difficulty in diagnosis, especially in animals in the subclinical stage.

The results of the present study confirmed the occurrence of ovine PTB in Khartoum North Locality, Khartoum State, the Sudan. Countrywide investigations would reveal the prevalence of the disease in other states. Besides being a production-limiting disease, it is gaining more public health importance as the association between MAP and some human diseases is garnering more support [7,34]. 

## 4. Conclusions

In conclusion, all tests used here confirmed the diagnosis of paratuberculosis in the sheep flock, but the molecular technique was superior. In the absence of advanced techniques, typical acid-fast bacilli of MAP are almost diagnostic. Reports of paratuberculosis in sheep warrant special considerations for further investigations on this disease in the country for planning control programmes for PTB. 

## Figures and Tables

**Table 1:** Detection of *Mycobacterium avium* subsp. *paratuberculosis* DNA by recombinase polymerase amplification (RPA) in relation to arrangements of acid-fast bacilli in faecal smears of sheep.

Acid Fast Bacilli		RPA Tested	
Arrangement of Bacilli	Number	Number	Positive (%)
Many scattered	24	12	6 (50)
Few scattered	28	16	2 (12.5)
Many clumps	3	2	2 (100)
Few clumps	3	2	2 (100)
Total	58	32	12

**Table 2:** Screening of *Mycobacterium avium* subsp. *paratuberculosis* in faecal samples of sheep by different diagnostic tests.

	Samples	Positive	
		N	%
ZN staining	59	58	98.3
ELISA	59	3	5.1
RPA	32	12	37.5
Culture	6	2	33.3
